# Comparative Pathogenicity of United Kingdom Isolates of the Emerging Pathogen *Candida auris* and Other Key Pathogenic *Candida* Species

**DOI:** 10.1128/mSphere.00189-16

**Published:** 2016-08-18

**Authors:** Andrew M. Borman, Adrien Szekely, Elizabeth M. Johnson

**Affiliations:** UK National Mycology Reference Laboratory (MRL), Public Health England South-West, Bristol, United Kingdom; Carnegie Mellon University

**Keywords:** pathogenicity, *Candida auris*, pathogenic yeasts, emerging pathogen

## Abstract

*Candida auris*, first described in 2009, has since emerged as an important, multidrug-resistant, nosocomial agent of candidemia, with large outbreaks reported worldwide and high mortality rates associated with therapeutic failure. The current study employed *C. auris* isolates from a variety of centers in the United Kingdom to evaluate the pathogenicity of this emerging pathogen compared to that of other common pathogenic yeast species in the invertebrate *Galleria mellonella* infection model. We showed that *C. auris* isolates differ in their growth characteristics *in vitro*, with a proportion of isolates failing to release daughter cells after budding, resulting in the formation of large aggregates of cells that cannot be physically disrupted. Our results also demonstrate strain-specific differences in the behavior of *C. auris* in *G. mellonella*, with the aggregate-forming isolates exhibiting significantly less pathogenicity than their nonaggregating counterparts. Importantly, the nonaggregating isolates exhibited pathogenicity comparable to that of *C. albicans*, which is currently accepted as the most pathogenic member of the genus, despite the fact that *C. auris* isolates do not produce hyphae and produce only rudimentary pseudohyphae either *in vitro* or in *G. mellonella*.

**IMPORTANCE** The incidence of invasive candidiasis, which includes candidemia and deep tissue infections, continues to rise and is associated with considerable mortality rates. *Candida albicans* remains the most common cause of invasive candidiasis, although the prevalence of non-*albicans* species has increased over recent years. Since its first description in 2009, *Candida auris* has emerged as a serious nosocomial health risk, with widespread outbreaks in numerous hospitals worldwide. However, despite receiving considerable attention, little is known concerning the pathogenicity of this emerging fungal pathogen. Here, using the *Galleria mellonella* insect systemic infection model, we show strain-specific differences in the virulence of *C. auris*, with the most virulent isolates exhibiting pathogenicity comparable to that of *C. albicans*, which is currently accepted as the most pathogenic member of the genus.

## Abstract (precis)

The incidence of invasive candidiasis, which includes candidemia and deep tissue infections, continues to rise and is associated with considerable mortality rates. *Candida albicans* remains the most common cause of invasive candidiasis, although the prevalence of non-*albicans* species has increased over recent years. Since its first description in 2009, *Candida auris* has emerged as a serious nosocomial health risk, with widespread outbreaks in numerous hospitals worldwide. However, despite receiving considerable attention, little is known concerning the pathogenicity of this emerging fungal pathogen. Here, using the *Galleria mellonella* insect systemic infection model, we show strain-specific differences in the virulence of *C. auris*, with the most virulent isolates exhibiting pathogenicity comparable to that of *C. albicans*, which is currently accepted as the most pathogenic member of the genus.

## INTRODUCTION

The incidence of invasive fungal infections caused by unusual *Candida* spp. continues to rise, driven in part by increased populations of immunocompromised patients and those undergoing invasive procedures (1 – 8). However, to date, *Candida albicans* remains the most frequently isolated *Candida* species in the clinical setting, is the principal agent of nosocomial yeast infections (1, 4 – 6), and is widely accepted as being the most pathogenic *Candida* species (reviewed in references 9 and 10).

In 2009, a novel *Candida* species in the *Candida haemulonii* complex (*Metchnikowiaceae*), *Candida auris*, was described after isolation from a discharge from a human external ear canal in Japan (11). Subsequent studies confirmed an association with chronic otitis media in 15 patients from South Korea (12), with evidence of clonal transmission and resistance to certain triazole antifungal agents. *C. auris* has since been reported from a wide spectrum of clinical manifestations, ranging from colonization through deep-seated infections and candidemia (13 – 17). Today, it is evident that *C. auris* has emerged as an important nosocomial pathogen with clonal inter- and intrahospital transmission, and it has become widespread across several Asian countries and South Africa (13 – 18). *C. auris* fungemia is associated with a high mortality rate, therapeutic failure (13 – 15), and widespread resistance to several classes of antifungal agents (13, 15 – 21). Furthermore, correct identification of *C. auris* isolates is complicated by the fact that many commercially available biochemical-based tests can misidentify *C. auris* as the phylogenetically related species *Candida haemulonii* (11, 12, 19 – 23), which presents an additional challenge for appropriate patient management.

The first 2 United Kingdom isolates of *C. auris* were received at the UK National Mycology Reference Laboratory (MRL) in 2013, from blood cultures from 2 unrelated patients in distant geographical localities (MRL unpublished data). Since 2013, we have received a further 19 isolates from at least 6 different hospitals, including 14 isolates suspected of being part of an outbreak. Here we have compared the pathogenicities of 12 United Kingdom isolates of *C. auris* from 6 different referring National Health Service (NHS) hospitals with the pathogenicities of equivalent isolates of other common pathogenic *Candida* species, using the *Galleria mellonella* insect systemic infection model.

## RESULTS AND DISCUSSION

**FIG 1:**
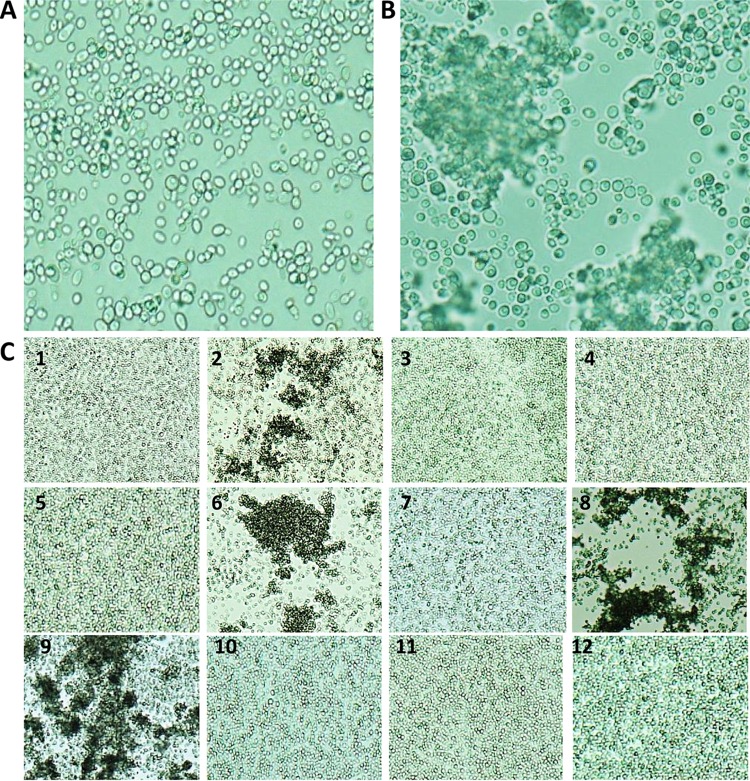
Microscopic appearance of non-aggregate-forming isolates (A) and aggregate-forming isolates (B) of *C. auris* in PBS suspensions. Suspensions were subjected to vortex mixing for 1 min prior to examination at ×1,000 magnification. (C) The 12 isolates of *C. auris* employed in the current study (×100 magnification).

The characteristics of the 12 isolates of *C. auris* employed in the current study are detailed in Table 1, with antifungal MIC values determined at the MRL. Initial attempts to generate suspensions of *C. auris* isolates in phosphate-buffered saline (PBS) for larval inoculation revealed striking strain-specific differences in phenotypic behavior. While most isolates readily formed homogeneous suspensions upon thorough vortex mixing, the resulting suspensions seen with 4 independent isolates from 3 different referring hospitals were grossly particulate and contained individual yeast cells mixed with large aggregations (“aggregate” strains) (Table 1 and Fig. 1). For these 4 isolates, aggregates could not be physically disrupted by vigorous vortex mixing or by detergent treatments (data not shown). Since the aggregates were too large to permit larval inoculation and since cell numbers within the aggregates could not be accurately quantified, homogeneous suspensions were instead achieved by allowing initial suspensions to settle for 10 min, followed by removal of the supernatant containing individual yeast cells that had remained in suspension and adjustment of these individual cells to the appropriate concentration for injection into larvae.

**TABLE 1:** Origin of the *Candida auris* strains employed in this study^a^

Isolate	Yr	Site	Hospital	Morphology	MIC value (mg/liter)					
					AMB	FLC	VRC	PSC	ANID	5FC
1	2015	Sputum	A	Single cells	0.5	8	0.06		0.25	
2	2015	CSF	B	Aggregates	0.5	>64	0.5	<0.03		<0.125
3	2015	Not stated	A	Single cells	1.0	8	0.06		0.25	
4	2015	Line	A	Single cells	0.5	8	0.06		0.03	
5	2015	Arterial line	A	Single cells	0.5	16	0.125		0.5	
6	2014	Pleural fluid	C	Aggregates	0.5	>64	0.25		0.125	
7	2016	Not stated	D	Single cells	1	16	0.125		0.25	
8	2015	Pustule swab	B	Aggregates		>64				
9	2016	Blood culture	E	Aggregates	0.5	64	2		0.125	
10	2016	Wound swab	F^b^	Single cells	1	>64	16		0.25	
11	2015	Femoral line	A	Single cells	0.5	8	0.06		0.06	
12	2016	Not stated	E	Single cells	0.5		0.5	1.0		0.25

a The antifungal susceptibility results expressed as MICs (in milligrams per liter) are given for those antifungal agents requested by referring centers; the susceptibility tests were performed at the MRL. MICs were obtained using CLSI broth microdilution methodologies (26). Abbreviations: AMB, amphotericin B; FLC, fluconazole; VRC, voriconazole; PSC, posaconazole; ANID, anidualfungin; 5FC, flucytosine; CSF, cerebrospinal fluid.

b The patient was transferred from hospital A.

**FIG 2:**
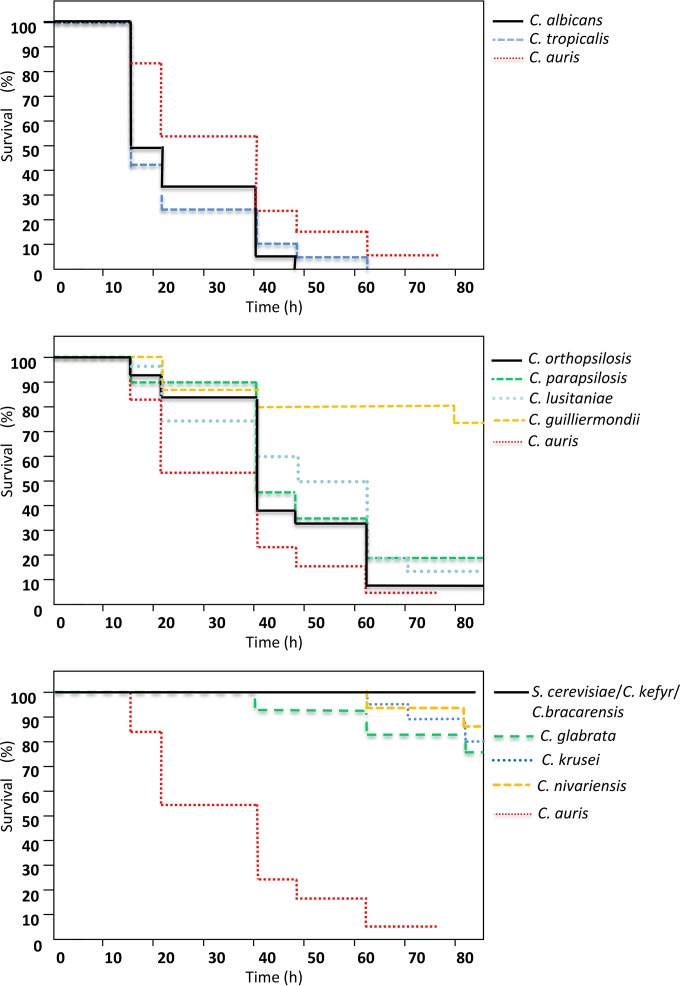
The virulence of *Candida* species in *Galleria mellonella* larvae at 37°C is species specific. Kaplan-Meier plots of *G. mellonella* survival after injection with 10^6^ CFU/larva of the indicated *Candida* species, organized as those that produce true hyphae (top panel), pseudohyphae (middle panel), or no hyphae/pseudohyphae (bottom panel), are shown. Equivalent plots obtained with *C. auris* isolates are included in all three panels for comparison. Four strains were tested per species, with 15 larvae per strain (60 larvae per species), except for *C. auris*, where 12 strains were included, with 10 larvae per strain. Experiments were performed in duplicate; plots represent the combined (additive) data from all strains and all experiments. No larval killing was observed in control larvae injected with an equivalent volume of PBS.

In agreement with previous reports (10, 24), the pathogenicity of the common *Candida* species at 37°C in *G. mellonella* was directly related to the ability of individual species to produce hyphal filaments or pseudohyphae (Fig. 2; see also Fig. S1 in the supplemental material), with very little strain-to-strain variation in virulence within each species (see Fig. S1). Thus, *C. albicans* and *C. tropicalis* exhibited greater virulence than *C. lusitaniae*, *C. guilliermondii*, and members of the *C. parapsilosis* species complex, and virtually no larval killing was induced by those organisms that form only rudimentary pseudohyphae or no pseudohyphae (*C. glabrata*, *C. nivariensis*, *C. krusei*, *C. kefyr*, *C. bracarensis*, and *Saccharomyces cerevisiae*) (Fig. 2; see Table 2 for full statistical analyses).

10.1128/mSphere.00189-16.1 Figure S1  Individual killing curves for the common *Candida* species depicted in Fig. 2, with error bars representing the maximum and minimum larval killing rates observed with different isolates of that species at each time point. Download Figure S1, TIF file, 0.05 MB. Copyright © 2016 Borman et al. 2016 Borman et al. This content is distributed under the terms of the Creative Commons Attribution 4.0 International license.

**TABLE 2:** Statistical analyses of species-specific differences in pathogenicity^a^

Species	Pathogenicity difference *P* value for species:														
	1	2	3	4	5	6	7	8	9	10	11	12	13	14	15
1. *C. albicans*															
2. *C. tropicalis*	ns														
3. *C. auris* (all)	ns	ns													
4. *C. auris* (single)	ns	ns	ns												
5. *C. auris* (aggregative)	0.008	0.007	ns	0.02											
6. *C. parapsilosis*	0.04	0.01	ns	0.04	ns										
7. *C. orthopsilosis*	0.04	0.03	ns	0.05	ns	ns									
8. *C. lusitaniae*	0.01	0.01	ns	0.04	ns	ns	ns								
9. *C. guilliermondii*	0.001	0.001	0.01	0.001	ns	ns	ns	0.02							
10. *C. glabrata*	0.001	0.001	0.001	0.001	0.01	0.01	0.01	0.01	0.02						
11. *C. krusei*	0.001	0.001	0.001	0.001	0.002	0.003	0.002	0.003	0.009	ns					
12. *C. nivariensis*	0.001	0.001	0.001	0.001	0.001	0.001	0.003	0.004	0.009	ns	ns				
13. *C. bracarensis*	0.001	0.001	0.001	0.001	0.001	0.001	0.001	0.001	0.007	0.03	ns	ns			
14. *C. kefyr*	0.001	0.001	0.001	0.001	0.001	0.001	0.001	0.001	0.007	0.03	ns	ns	ns		
15. *S. cerevisiae*	0.001	0.001	0.001	0.001	0.001	0.001	0.001	0.001	0.007	0.03	ns	ns	ns	ns	

a *P* values of <0.05 as determined using the Mann-Whitney (two-sample Wilcoxon) test are given for all species combinations where a given species (horizontal axis) was more pathogenic than another (vertical axis). ns, not statistically significant (*P* > 0.05). 1, *C. albicans*; 2, *C. tropicalis*; 3, *C. auris* (all); 4, *C. auris* (single); 5, *C. auris* (aggregative); 6, *C. parapsilosis*; 7, *C. orthopsilosis*; 8, *C. lusitaniae*; 9, *C. guilliermondii*; 10, *C. glabrata*; 11, *C. krusei*; 12, *C. nivariensis*; 13, *C. bracarensis*; 14, *C. kefyr*; 15, *S. cerevisiae*.

**FIG 3:**
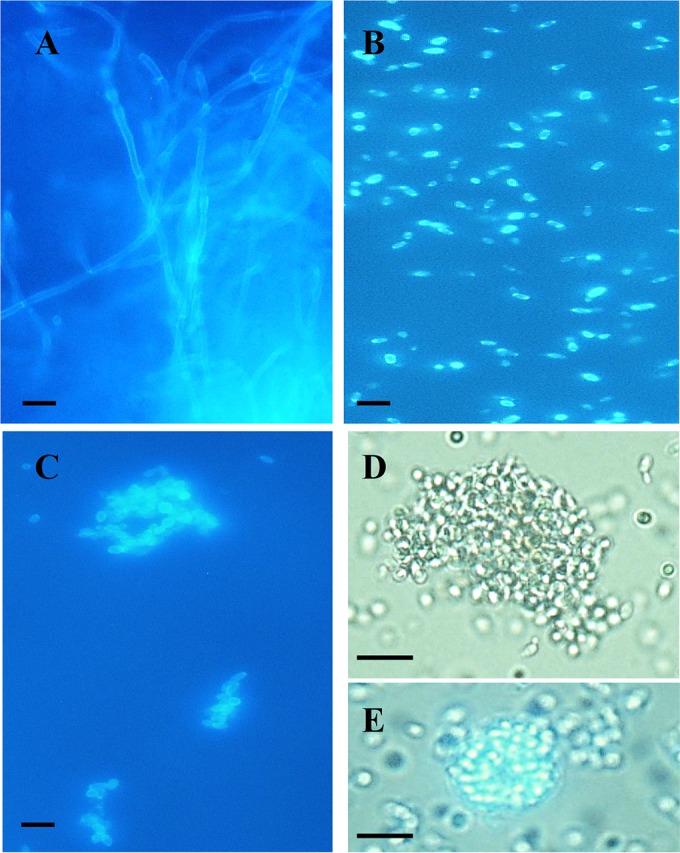
Microscopic appearance of hemolymph from infected larvae. (A to D) Hemolymph recovered after 18 h at 37°C in larvae inoculated with *C. albicans* (A), a nonaggregating strain of *C. auris* (strain 1) (B), and single cells prepared from an aggregate-forming isolate of *C. auris* (strain 2) (C and D). The hemolymph was stained with Calcofluor fluorescent enhancer after KOH treatment and examined under UV illumination (A to C) or was examined directly by light microscopy (D and E). Panel E shows a single phagocytic cell containing many individual budding *C. auris* cells. Magnification in all panels was ×400. Scale bar = 10 µm.

**FIG 4:**
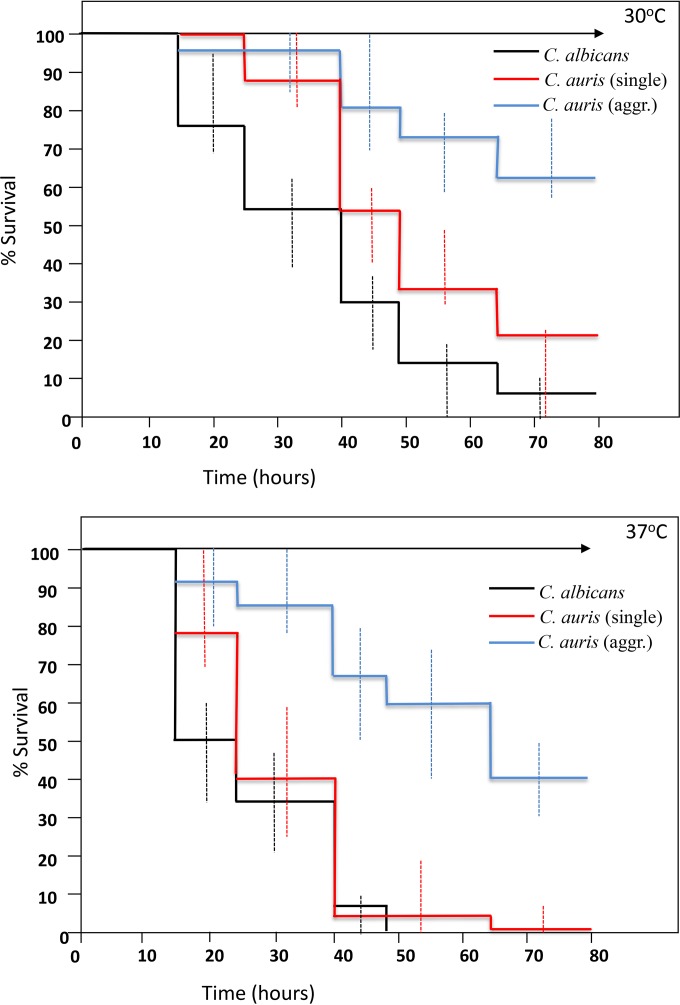
Virulence of aggregate-forming and nonaggregate strains of *Candida auris* compared to *C. albicans* in *Galleria mellonella* larvae at 30°C (upper panel) and 37°C (lower panel). Kaplan-Meier plots of *G. mellonella* survival after injection with 10^6^ CFU/larva of *Candida albicans* (black line), nonaggregating *C. auris* strains (red line), and aggregate-forming *C. auris* strains (blue line) are shown. Four strains were tested for *C. albicans*, with 15 larvae per strain, and 8 and 4 strains were tested for nonaggregate and aggregate-forming *C. auris*, respectively (with 10 larvae per strain). Experiments were performed in duplicate; plots represent the combined (additive) data from all strains and all experiments. Error bars represent the maximum and minimum larval killing observed with different isolates of each species at each time point. No larval killing was observed in control larvae injected with an equivalent volume of PBS (arrowed lines).

Strikingly, despite most reports suggesting that *C. auris* does not form significant pseudohyphae *in vitro* (14, 15, 21), *C. auris* strains exhibited virulence in *G. mellonella* that was significantly higher (in terms of the kinetics of larval death and the number of larvae killed) than that seen with most other common pathogenic yeast species, with overall pathogenicity approaching that observed with *C. albicans* and *C. tropicalis* isolates (Fig. 2 and Table 2). Dissection of representative larvae that had been inoculated with the various strains and incubated at 37°C for 18 h revealed significant hyphal proliferation in hemolymph form larvae inoculated with *C. albicans* (Fig. 3A). However, no hyphal or pseudohyphal formation was observed in larvae infected with any *C. auris* strains at 18 h or any time postinfection (Fig. 3B to D). Interestingly, in larvae that had received nonaggregating strains of *C. auris*, larval dissection revealed large numbers of individual budding yeast cells, including in phagocytic cells (Fig. 3B and E). However, in larvae inoculated with individual yeast cells prepared from aggregate-forming strains of *C. auris*, hemolymph contained large aggregates of *C. auris* cells, with few individual yeast cells, indicating that the ability to produce large aggregates had been maintained *in vivo* (Fig. 3C and E). In the light of this differential behavior of *C. auris* isolates in *G. mellonella*, further experiments compared larval killing with aggregate-forming versus non-aggregate-forming strains, with larvae incubated at both 30°C and 37°C. Strikingly, nonaggregate strains exhibited significantly greater virulence than aggregate-forming strains at both temperatures (Fig. 4 and Table 2) (*P* = 0.02), with nonaggregate isolates showing virulence that was indistinguishable from that of *C. albicans* strains at 37°C (Fig. 4).

In the current report, we present for the first time a comparative study of the pathogenicities of isolates of *Candida auris* and those of other common pathogenic *Candida* species and the somewhat surprising finding that *C. auris* virulence is comparable to that seen with *C. albicans* in the invertebrate *G. mellonella* model, despite the fact that *C. auris* isolates do not undergo significant filamentation in this model organism. This finding is all the more striking since *C. auris* yeast cells are more comparable in size and growth rate to *C. glabrata* than to *C. albicans* (Fig. 2 and data not shown). Moreover, we have demonstrated the novel finding that certain *C. auris* isolates form large aggregates of cells both *in vitro* and *in vivo* in inoculated larvae, even when larvae were inoculated with individual cells prepared from aggregating isolates. Microscopic examination of these aggregates suggests that they form due to reduced daughter cell liberation after budding (see, for example, Fig. 3C), rather than due to flocculation of individual budding cells. This contention would certainly be supported by our inability to disrupt the aggregates with intense vortex mixing and detergent treatments. In *G. mellonella*, aggregate-forming strains exhibit less virulence than those strains that exist as single budding cells. Further studies will be required to determine if aggregate-forming strains produce less dissemination during infections in humans or, conversely, whether the ability to form large aggregates protects those strains against phagocytic attack or the effects of antifungal agents or detergents used to clean hospital environments.

## MATERIALS AND METHODS

### Fungal strains.

All *C. auris* isolates were identified by ribosomal DNA (rDNA) gene sequencing targeting the 28S rRNA or by internal transcribed spacer 1 (ITS1) regions and matrix-assisted laser desorption ionization–time of flight (MALDI-TOF) analysis or by a combination of the two methods exactly as described previously (25). For the other *Candida* species included for comparison, where possible, clinical isolates were from deep-seated infections. Identity to the species level was confirmed by sequencing or MALDI-TOF analysis in all cases.

### Killing assays in *G. mellonella.*

Killing assays were performed in *Galleria mellonella* exactly as described previously (10), using final (sixth) instar larvae (Livefood UK Ltd., Rooks bridge, Somerset, United Kingdom) weighing approximately 300 mg each that were free of gray markings and that had been maintained at room temperature in the dark and inoculated within 48 h of receipt. Suspensions of individual *Candida* isolates that had been grown on Sabouraud’s agar for 24 h at 37°C were harvested by gentle scraping of colony surfaces with sterile plastic loops, washed twice in sterile PBS, counted in hemocytometers, and adjusted to 10^5^ cells/μl in sterile PBS. Individual larvae were inoculated in the left rear proleg with 1 × 10^6^ yeast cells–PBS (final inoculum volume, 10 µl) using a 10-µl Hamilton syringe fitted with a 26-gauge blunt needle. At least 10 larvae were inoculated per isolate per experiment (experiments employed 4 independent isolates of each *Candida* test species [12 isolates in the case of *C. auris*]). Control groups of larvae received 10 µl of sterile PBS in exactly the same manner. Inoculated larvae were incubated at 30°C or 37°C and scored for viability at 8-h intervals as described previously (10). Differences in resulting Kaplan-Meier survival plots were evaluated using the Mann-Whitney (two-sample Wilcoxon) test. In some experiments, fungal cell filamentation postinfection was assessed by sacrificing representative larvae from each inoculum group at 24 h postinfection and aseptic collection of the fat body/solid internal structures and hemolymph followed by microscopic examination (10).

### Antifungal susceptibility testing of *C. auris* isolates.

Broth microdilution determination of yeast MICs was performed according to CLSI method M27-A3 (26) in round-bottomed 96-well plates with yeast blastospore suspensions prepared in saline solution and then diluted into RPMI 1640 and adjusted to a final concentration of 2.5 × 10^3^ CFU/ml. Inoculated plates were incubated for 24 to 48 h at 35°C. MICs were read at 24 and 48 h as the concentration of drug that elicited 100% inhibition of growth (amphotericin B) or significant (approximately 50%) inhibition of growth compared with that of a drug-free control (fluconazole, voriconazole, posaconazole, anidulafungin, and flucytosine).
