# Supplementary figures and images for: Comparative Pathogenicity of United Kingdom Isolates of the Emerging Pathogen Candida auris and Other Key Pathogenic Candida Species
Source: mSphere. 2016 Aug 18;1(4):e00189-16. doi: 10.1128/mSphere.00189-16 (PMC4990711; doi:10.1128/mSphere.00189-16)

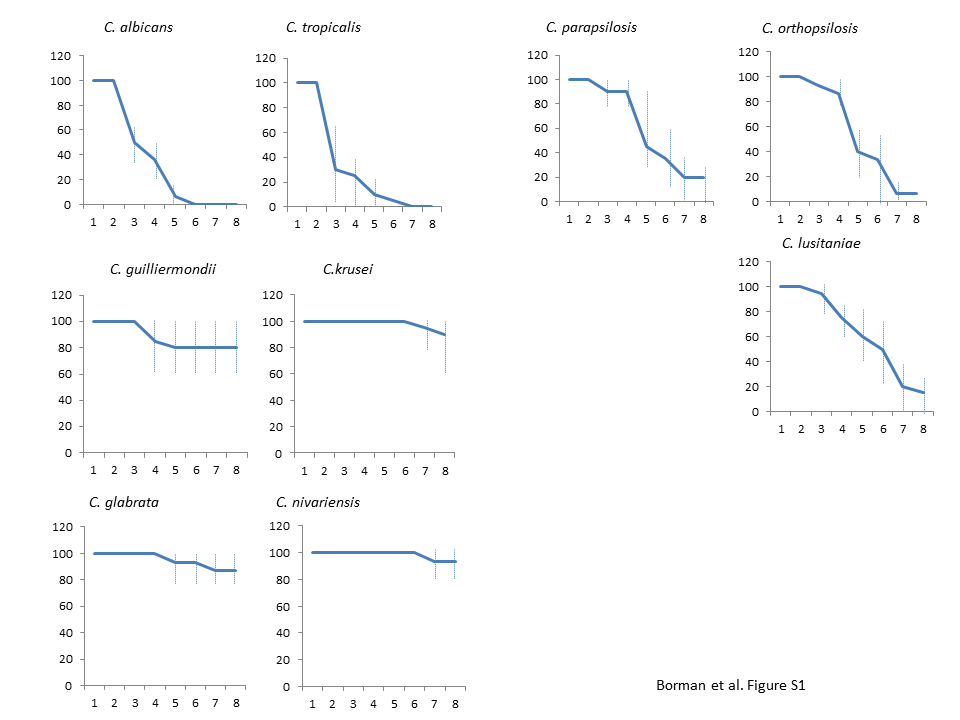

Supplement: Figure S1 [file sph004162137sf1.tif]
